# Development of a multi-epitope vaccine candidate to combat SARS-CoV-2 and dengue virus co-infection through an immunoinformatic approach

**DOI:** 10.3389/fimmu.2025.1442101

**Published:** 2025-02-26

**Authors:** Saurav Mandal, Waribam Pratibha Chanu, Kalimuthusamy Natarajaseenivasan

**Affiliations:** ^1^ Division of Metabolomics, Proteomics & Imaging facility, Regional Medical Research Centre, Indian Council of Medical Research (ICMR), Dibrugarh, Assam, India; ^2^ Department of Applied Physics, School of Vocational Studies and Applied Sciences (SoVSAS), Gautam Buddha University, Greater Noida, Uttar Pradesh, India

**Keywords:** SARS-CoV-2, dengue, multi-epitope vaccine, immunoinformatics, IEDB

## Abstract

**Background:**

Although the SARS-CoV-2 and dengue viruses seriously endanger human health, there is presently no vaccine that can stop a person from contracting both viruses at the same time. In this study, four antigens from SARS-CoV-2 and dengue virus were tested for immunogenicity, antigenicity, allergenicity, and toxicity and chosen to predict dominant T- and B-cell epitopes.

**Methods:**

For designing a multi-epitope vaccine, the sequences were retrieved, and using bioinformatics and immunoinformatics, the physicochemical and immunological properties, as well as secondary structures, of the vaccine were predicted and studied. Additionally, the three-dimensional structure was estimated, improved upon, and confirmed using bioinformatics methods before being docked with TLR-2 and TLR-4. Eight helper T-cell lymphocyte (HTL) epitopes, ten cytotoxic T-cell lymphocyte (CTL) epitopes, nine B-cell epitopes, and TLR agonists were used to create a new multi-epitope vaccine. Furthermore, according to the immunological stimulation hypothesis, the vaccine could stimulate T and B cells to create large quantities of Th1 cytokines and antibodies.

**Results:**

The study indicates that the developed vaccine is a favorable vaccine candidate with antigenicity, immunogenicity, non-toxicity, and non-allergenicity properties. The vaccine construct was made up of 460 amino acids, had an MW of 49391.51 Da, a theoretical pI of 9.86, and the formula C_2203_H_3433_N_643_O_618_S_18_, a lipid index of 39.84, a GRAVY of −0.473, an aliphatic index of 63.80, and an instability index of 39.84, which classifies the protein to be stable.

**Conclusion:**

The acquired data showed that both vaccine designs had a considerable chance of preventing the co-infection of SARS-CoV-2 and dengue virus and that they demonstrate good results following *in-silico* testing. Furthermore, the vaccine may be an effective strategy in preventing SARS-CoV-2 and dengue since it can cause noticeably high levels of Th1 cytokines and antibodies.

## 1 Introduction

The beta genus of coronaviruses includes the Severe Acute Respiratory Syndrome Coronavirus 2 (SARS-CoV-2) that leads to the loss of millions of lives across the globe. WHO has received reports of 775.91 million confirmed cases of COVID-19, including 7.05 million deaths as of 11 August 2024 (https://data.who.int/dashboards/covid19/cases). The human SARS-CoV virus that led to the SARS outbreak in 2002–2004 is proximally related to the SARS-CoV-2. Coronaviruses, like most RNA viruses, undergo rapid genetic changes over months or years, making these mutations observable and measurable (1). COVID-19 is not limited to particular racial, gender, or age groups. However, some underlying medical issues and co-infection are regarded as risk factors and are linked to greater mortality rates (2). Many vaccines are currently in preclinical and clinical development, and several COVID-19 vaccines, such as ChAdOx1 nCoV-19, BBV152/Covaxin, NVX-CoV2373, and so forth, have been approved for the prevention of COVID-19 (3–8). Dengue virus, which has four distinct serotypes, is transmitted primarily by the Aedes aegypti and Aedes albopictus mosquitoes. This virus is responsible for causing one of the most severe vector-borne diseases, particularly in tropical and subtropical regions around the world. The spread of dengue has become a significant public health concern, as the virus can lead to a range of symptoms, from mild fever to severe dengue, which can be life threatening (9–11). Aedes aegypti, the primary vector, is a diurnal mosquito that thrives in peridomestic environments, capable of biting multiple individuals in quick succession and breeding in various manmade containers that collect water. Aedes mosquitoes can spread more widely due to global warming, which raises the risk of dengue epidemics in temperate areas (12). CTD, Dengvaxia developed by Sanofi-Pasteur, is the first available vaccine for dengue (10, 13). Some candidate vaccines for dengue in the clinical trial phase are TV003/TV005, DENVax, monovalent DENV-1, tetravalent prM/E, V180, and TLAV-prime/PIV-boost (10, 14–17). In countries with relatively poor healthcare infrastructure, dengue is often endemic or hyperendemic (circulation of many DENV serotypes). The simultaneous outbreaks of these diseases posed a serious threat because they could be devastating for the healthcare systems in these nations, particularly in cases of co-infection by SARS-CoV-2 and DENV. This was especially true given the stress caused by the COVID-19 pandemic (11). In several case studies conducted across South America, South Africa, and South Asia, the co-infection of SARS-CoV-2 and dengue virus has been extensively documented, underscoring the immense burden it imposes on public health systems. For example, in South America, studies by Bicudo et al. (18) and Carosella et al. (19) reported cases of simultaneous infections in Brazil and Argentina. These reports highlighted the challenges faced by healthcare professionals in managing patients presenting with overlapping symptoms such as fever, headache, and thrombocytopenia, common to both diseases. Similarly, in South Africa, Epelboin et al. (20) and Verduyn et al. (21) detailed instances of co-infection, particularly in regions already grappling with endemic dengue outbreaks, where the added burden of SARS-CoV-2 stretched healthcare resources to their limits.

In South Asia, the situation has been equally concerning (22). Case studies from countries like India, Pakistan, and the Philippines have revealed multiple instances of co-infection (23–25). For instance, Mahajan et al. (25) documented co-infections during pregnancy, which increased the risk of complications and adverse maternal outcomes. Similarly, Saddique et al. (26) and Saipen et al. (27) highlighted the diagnostic and treatment challenges in regions where dengue is hyperendemic and the healthcare infrastructure is relatively underdeveloped. These studies emphasized that the co-circulation of SARS-CoV-2 and dengue exacerbates the strain on healthcare systems already overwhelmed by the pandemic.

Despite differences in the pathogenesis of these viruses, one being an RNA respiratory virus and the other a mosquito-borne flavivirus, their simultaneous infections often result in overlapping clinical presentations, including fever, headache, nausea, myalgia, and thrombocytopenia (28). Such similarities make it extremely challenging to differentiate between the two diseases, especially in resource-limited settings where diagnostic tools may not be readily available. The result is a heightened risk of misdiagnosis, delayed treatment, and suboptimal patient outcomes. For instance, patients may be misdiagnosed with only one of the infections, leading to inadequate care for the other.

The dual burden of these diseases highlights the urgent need for innovative strategies to mitigate their impact. One crucial approach is the development of multi-epitope vaccines that can provide cross-protection against both SARS-CoV-2 and dengue. Such vaccines would not only reduce the risk of co-infection but also alleviate the diagnostic and management challenges posed by overlapping epidemics, particularly in regions where both viruses are endemic. These vaccines represent a proactive and comprehensive strategy to strengthen global health resilience in the face of emerging infectious diseases.

The goal of this research is to create a novel, multi-epitope vaccine against SARS-CoV-2 and dengue viruses. In this study, a multi-epitope vaccine was developed by incorporating the surface spike, membrane, and nucleocapsid proteins from SARS-CoV-2, along with the genome polyprotein from the dengue virus. The vaccine’s physicochemical properties, secondary structures, and three-dimensional (3D) structure were predicted and refined, and it was docked with TLR-2 and TLR-4. A potential vaccine has been designed by leveraging diverse immunoinformatics techniques to integrate several epitopes from both SARS-CoV-2 and dengue virus. This vaccine includes a range of helper T-cell, B-cell, and CTL epitopes that are strategically chosen to stimulate robust cellular and humoral immune responses. The vaccine demonstrated high antigenicity, immunogenicity, non-toxicity, and non-allergenicity, with a molecular weight of 49,391.51 Da and a stability index of 39.84. By targeting key viral proteins from SARS-CoV-2 and dengue, the vaccine aims to induce a comprehensive immune defense. We propose that these carefully selected epitopes could represent a promising candidate for developing an effective vaccine to combat infections caused by both SARS-CoV-2 and dengue virus, offering a potential strategy for addressing co-infections and enhancing public health protection.

## 2 Materials and methods

### 2.1 Identification of target antigen

Due to their significant role in immune response, the SARS-CoV-2 surface spike (NCBI Accession number: QHR63290.1), membrane (NCBI Accession number: QHR63293.1), and nucleocapsid (NCBI Accession number: QHR63298.1) proteins were chosen as prospective antigens for epitope prediction (29–31). For the dengue virus, the genome polyprotein (Uniprot ID: P29991.1) was selected as the antigen. This polyprotein encompasses all structural [capsid (C), pre-membrane (prM), and envelope (E)] and non-structural (NS1, NS2A, NS2B, NS3, NS4A, NS4B, and NS5) proteins of the virus. The entire polyprotein sequence was used to predict potential epitopes, ensuring a broad range of targets for the immune response (32, 33). Full-length amino acid sequences were retrieved from the National Center for Biotechnology Information (NCBI) and Uniprot databases. In our study, we selected specific proteins from SARS-CoV-2 and dengue virus for the prediction of cytotoxic T-lymphocyte (CTL) epitopes, helper T-cell lymphocyte (HTL) epitopes, and linear B-cell epitopes to enhance the effectiveness of our vaccine design. For SARS-CoV-2, we focused on three key proteins: the surface spike protein (QHR63290.1), the membrane glycoprotein (QHR63293.1), and the nucleocapsid protein (QHR63298.1). The surface spike protein is crucial for the virus’s entry into host cells by binding to the ACE2 receptor, making it a prime target for immune responses. The membrane glycoprotein plays a vital role in the assembly and release of new viral particles, while the nucleocapsid protein is essential for packaging viral RNA and stabilizing the nucleocapsid, thus supporting viral replication and stability. From the dengue virus, we included the genome polyprotein (ID: P29991.1), a precursor that is processed into several functional proteins critical for viral replication and assembly. This polyprotein’s role in forming viral components and interacting with the immune system makes it an important target for epitope prediction. The decision to focus on these proteins rather than all available proteins in the dataset was driven by their significant roles in pathogen virulence, surface exposure, and their potential to elicit strong immune responses. Proteins that are surface-exposed or secreted are prioritized as they are more likely to interact with the immune system and be recognized as foreign, which is essential for an effective immune response. Focusing on these specific proteins allows for a targeted and efficient prediction process, ensuring that the identified epitopes are those most likely to contribute to a potent immune response. This approach enhances the specificity of the epitope prediction and improves the overall efficiency of vaccine design by concentrating on proteins with high antigenic potential. By strategically selecting and analyzing these proteins, our study aims to identify the most promising epitopes for robust antibody production, thus optimizing the development of an effective vaccine candidate.

### 2.2 Prediction of immunodominant CTL epitopes

Identifying cytotoxic T lymphocyte (CTL) epitopes is crucial for developing an effective multi-epitope vaccine. To predict these epitopes, we utilized the NetCTL v1.2 online server, which evaluates peptide sequences for their potential to bind to MHC Class I molecules. CD8+ CTL epitopes are typically 9–10 amino acids long and are recognized by MHC Class I molecules. This server integrates multiple prediction models, including MHC Class I binding affinity, proteasomal cleavage, and TAP transport efficiency. By assessing these factors, NetCTL v1.2 provides a combined score indicating the likelihood of each peptide being presented by MHC Class I molecules and eliciting a CTL response. This approach ensures that only high-quality epitopes with strong potential to stimulate cytotoxic T-cells are selected for further evaluation (34, 35). The predicted CTL epitopes are presented in **Table 1**.

**Table 1:** CTL epitopes considered using the NetCTL along with their scores.

S. No	Peptide sequence	Virus	Protein	Net CTL score
1	ITEAELTGY	Dengue	Genome polyprotein	2.7278
2	MTDDIGMGV	Dengue	Genome polyprotein	2.2165
3	RVAAEGINY	Dengue	Genome polyprotein	1.2975
4	LSPVRVPNY	Dengue	Genome polyprotein	1.1299
5	GAAAYYVGY	SARS-CoV-2	Spike	1.2194
6	VLKGVKLHY	SARS-CoV-2	Spike	0.8253
7	QLTPTWRVY	SARS-CoV-2	Spike	0.7887
8	VLPFNDGVY	SARS-CoV-2	Spike	0.7675
9	ATSRTLSYY	SARS-CoV-2	Membrane	2.6146
10	LVGLMWLSY	SARS-CoV-2	Membrane	1.3974

### 2.3 Prediction of immunodominant HTL epitopes

To identify immunodominant HTL epitopes, we focused on key proteins from SARS-CoV-2 and dengue virus: the surface spike protein (QHR63290.1), membrane glycoprotein (QHR63293.1), nucleocapsid protein (QHR63298.1), and genome polyprotein (ID: P29991.1). These proteins were selected for their crucial roles in viral infection and their potential to elicit robust immune responses.

The length of 15 amino acids was chosen for epitope prediction because it is ideal for binding with Major Histocompatibility Complex (MHC) Class II molecules, which is essential for the activation of CD4+ T cells. This peptide length is widely documented to provide effective MHC Class II binding and presentation, making it a preferred choice for identifying potential epitopes (36–38). The selection of 15-mers ensures that the peptides are sufficiently long to be processed and presented on the surface of APCs, facilitating recognition by CD4+ T cells. The predicted HTL epitopes are shown in **Table 2**.

**Table 2:** HTL epitopes with IFN-γ positives.

S. No	Peptide sequence	Virus	Protein	Start	End	Method	Results IFN-γ
1	RKRRLTIMDLHPGAG	Dengue	Genome polyprotein	1659	1673	SVM	+
2	GCVVSWKNKELKCGS	Dengue	Genome polyprotein	778	792	SVM	+
3	FRKRRLTIMDLHPGA	Dengue	Genome polyprotein	1658	1672	SVM	+
4	TAGAAAYYVGYLQPR	SARS-CoV-2	Spike	259	273	SVM	+
5	QRVAGDSGFAAYSRY	SARS-CoV-2	Membrane	185	199	SVM	+
6	AQFAPSASAFFGMSR	SARS-CoV-2	Nucleocapsid	305	319	SVM	+
7	DAALALLLLDRLNQL	SARS-CoV-2	Nucleocapsid	216	230	SVM	+
8	IGYYRRATRRIRGGD	SARS-CoV-2	Nucleocapsid	84	98	SVM	+

For the prediction of these epitopes, the IEDB MHC Class II prediction tool was employed (http://tools.iedb.org/mhcii/). This tool generates a percentile rank for each peptide, reflecting its binding affinity to MHC Class II molecules. To ensure the identification of high-quality epitopes, only those with a percentile rank in the top 10%–20% were selected. This ranking system indicates that these peptides have a high likelihood of being presented by MHC Class II molecules, thus stimulating a robust CD4+ T-cell response. By using this criterion, we aimed to identify the most promising epitopes for inclusion in vaccine development, enhancing the specificity and efficacy of the immune response (39, 40).

### 2.4 Prediction of immunodominant linear B-cell epitopes

For linear B-cell epitopes, surface-exposed or secreted proteins are particularly prioritized because they are more likely to be recognized by the immune system as foreign, which is essential for effective antibody production. To predict linear B-cell epitopes, we utilized the ABCpred server (http://www.imtech.res.in/raghava/abcpred/), which applies a recurrent neural network model to identify potential epitopes (41). Linear B-cell epitopes are crucial for eliciting a humoral immune response, leading to the production of antibodies. The ABCpred server helps in identifying epitopes that are most likely to provoke a strong antibody response by analyzing the antigenicity of the selected proteins (42–44). The predicted linear B-cell epitopes are shown in **Table 3**.

**Table 3:** B-cell epitopes with their start position and predicted scores.

S. No	Peptide sequence	Virus	Protein	Start position	Predicted score
1	KGKRIEPSWADVKKDL	Dengue	Genome polyprotein	1536	0.95
2	PETAECPNTNRAWNSL	Dengue	Genome polyprotein	913	0.94
3	HGTIVIRVQYEGDGSP	Dengue	Genome polyprotein	597	0.94
4	GVSVITPGTNTSNQVA	SARS-CoV-2	Spike	594	0.95
5	GWTAGAAAYYVGYLQP	SARS-CoV-2	Spike	257	0.95
6	TRRIRGGDGKMKDLSP	SARS-CoV-2	Nucleocapsid	145	0.94
8	KSAAEASKKPRQKRTA	SARS-CoV-2	Nucleocapsid	303	0.93
9	TGSNQNGERSGARSKQ	SARS-CoV-2	Nucleocapsid	78	0.91

### 2.5 Prediction of immunodominant interferon–gamma positive HTL epitopes

Using the (15-mer) interferon–gamma (IFN-γ) epitope server, considered HTL epitopes were subjected to testing their ability to trigger an IFN-γ immune response. The IFN-γ positive epitopes are predicted by the server from overlapping sequences and the Support Vector Machine (SVM) methodology (45). Finally, the epitopes that produced a favorable IFN-γ response were chosen for the computational vaccine design.

### 2.6 Prediction of antigenicity, allergenicity, and toxicity of protein sequences

An essential component of vaccine development is ensuring that the potential vaccine candidates are antigenic. Only conceivable antigen epitopes were used in the creation of the vaccine. The antigenic assessment of considered sequences was carried out using a web server, VaxiJen 2.0, with a threshold value of 0.4 (46). In order to figure out which proteins are antigenic, the VaxiJen algorithm examines their physiochemical properties, which is mostly based on the approach of sequence alignment (47).

In this study, the allergenic qualities of epitopes were assessed using AllerTOP v.2.0 (http://www.ddg-pharmfac.net/AllerTOP) and AllergenFP (http://ddg-pharmfac.net/AllergenFP/). By investigating physiochemical characteristics of proteins, the web server AllerTOP v2.0 produced an accuracy of 85.3% during fivefold cross-validation (48). In contrast, AllergenFP is a descriptor and non-alignment fingerprint method to identify allergens and non-allergens (49). Epitopes that exhibited non-allergen characteristics were considered for future examination. Last, the ToxinPred web server (https://webs.iiitd.edu.in/ragha va/toxinpred/multisubmit.php) was considered to test the toxicity of each epitope, and non-toxic epitopes were selected (50).

### 2.7 Construction of the multi-epitope vaccine

**Figure 1:**
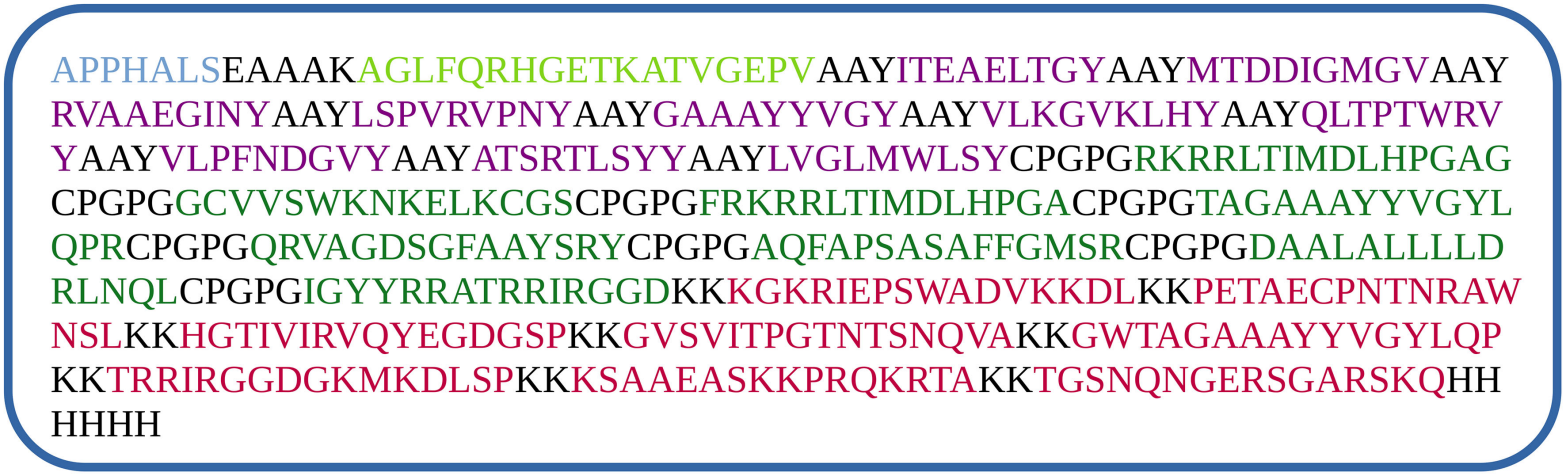
Vaccine construction using CTL (violet), HTL (dark green) and B-cell (red) epitopes using various linkers, RS-09 (light blue at the beginning), and PADRE (light green) sequence.

AAY, GPGPG, and KK linkers were utilized to combine immunodominant CTL, HTL, and B-cell epitopes to make a novel multi-epitope vaccine. To boost the vaccine’s immunogenicity and antigenicity, the toll-like receptor 4 (TLR4) agonist RS-09, 13 amino acid long non-natural pan DR epitope (PADRE), and a six-histamine tag sequence (HHHHH) were all added to the multi-epitope’s amino and carboxyl termini, respectively (51–53). The epitopes were linked to PADRE and TLR agonists via an EAAAK linker (refer to **Figure 1**).

### 2.8 Evaluation of the multi-epitope vaccine’s physicochemical characteristics and solubility

The Expasy Protparam server (https://web.expasy.org/cgi-bin/protparam/protparam) predicted the physicochemical properties of epitopes, including various parameters such as theoretical isoelectric point (pI), MW, amino acid constitution, non-stability, and grand average of hydropathicity (GRAVY), etc. (54, 55). Using the Protein-Sol service, the solubility of the multi-epitope vaccine was predicted to be 0.522 (56).

### 2.9 Prediction, optimization, and verification of secondary structure and three-dimensional structure

In this study, the Prabi and PSIPRED programs were chosen to forecast the secondary structures of the constructed protein. The Garnier-Osguthorpe-Robson 4 (GOR4) approach, which is used by the Prabi server to examine the secondary structure of peptides, has a shown accuracy of roughly 64.4% (57). It was used to additionally estimate secondary structure properties. The online server secondary structure generation program PSIPRED accurately predicts the alpha helix, beta strands, and coil, among other things (58). The I-TASSER (Iterative Threading ASSEmbly Refinement) server was used to create the multi-epitope vaccine’s tertiary or 3D model (59). By using iterative structural assembly simulations and multi-threaded comparisons, it creates three-dimensional atomic models of the protein (60). In addition to I-TASSER, we have also predicted the 3D structure of the vaccine candidate using Alphafold2 (61) and ESMfold (62). Then, the 3D vaccine model produced was refined utilizing the GalaxyRefine online server. When this technique is utilized to enhance the models created by cutting-edge protein structure prediction servers, both global and local structures’ quality can be improved (63). The protein 3D structure was validated using the ProSA-web server (https://prosa.services.came.sbg.ac.at/prosa.php). Based on the precise input structure, this program determines a total quality score and displays it as a Z score (64). High-resolution crystallographic structures were predicted using the ERRAT server (http://services.mbi.ucla.edu/ERRAT/), which was also utilized to examine non-bonded atom-atom interactions. By showing the proportion of residues in prohibited and permitted regions, a Ramachandran plot was retrieved from the SWISSPROT server to assess the quality of the modeled structure (65).

### 2.10 Prediction of conformational B-cell epitopes

There are discontinuous and linear B-cell epitopes, with the majority being the latter. As a result, anticipating conformational B-cell epitopes is essential to improving the spatial organization of the potential vaccines. A popular conformational B-cell epitope prediction server, ElliPro (http://tools.iedb.org/ellipro/), was chosen for the prediction with default parameters. The discontinuous B-cell epitopes that had scores over the threshold were established and used to create the vaccine (66).

### 2.11 TLRs and the multi-epitope vaccine’s molecular docking

Utilizing molecular docking to evaluate the interaction and coherence of binding between vaccines and human TLRs is a useful technique. TLR2 (PDB ID: 6NIG) and TLR4 (PDB ID: 4G8A) Protein Data Bank (PDB) data were first retrieved from the NCBI Molecular Modeling Database (MMDB). A ligand-receptor docking investigation was conducted using the internet server ClusPro 2.0 (https://cluspro.bu.edu/login.php) (67).

### 2.12 Prediction of the multi-epitope vaccine’s impact on Normal Mode Analysis and immunological responses

The iMODS web server (http://imods.Chaconlab.org/) was utilized to assess the structural dynamics and flexibility of the multi-epitope vaccine (68). The iMODS server uses Normal Modal Analysis (NMA) to exhibit the coordinated motion of the protein complex in internal coordinates (69). Special concepts like the B-factor, deformability, covariance, elastic model, and eigenvalues make up the evaluation parameters. Using the C-ImmSim server (http://150.146.2.1/C-IMMSIM/index.php), potential immunological reactions were estimated. The C-ImmSim online platform performs exceptionally well in foretelling the cellular and humoral reactions the vaccine induces in the human immune system (70). The C-ImmSim server performs exceptionally well in foretelling the cellular and humoral reactions the vaccine induces in the human immune system.

## 3 Results

### 3.1 Construction of novel multi-epitope

In designing the multi-epitope vaccine, we selected three key proteins from SARS-CoV-2: the surface spike protein, the membrane glycoprotein, and the nucleocapsid protein, due to their critical roles in viral infection and immune system interaction. For the dengue virus, we chose the genome polyprotein for its significance in viral assembly and immune response elicitation.

In the epitope selection process, we applied a binding affinity cutoff of >0.5 for both CD4+ T-cell (HTL) and CD8+ T-cell (CTL) epitopes to ensure robust MHC interactions. The final vaccine, “S10D16,” integrates eight HTL (**Table 2**), ten CTL (**Table 1**), and nine linear B-cell epitopes (**Table 3**) that successfully passed allergenicity, antigenicity, and toxicity tests. To enhance the vaccine’s immunogenicity, we used GGPPG, AAY, and KK linkers for the epitopes, while the PADRE helper peptide and TLR4 agonist RS-09 were connected using the EAAAK linker, thereby amplifying both the antigenic response and overall efficacy of the vaccine. HTL epitopes are high-binding MHC-II epitopes for human alleles HLA-DR that were predicted using the IEDB MHC-II online server. We selected 15 amino acids for epitope prediction because this length is optimal for binding with MHC Class II molecules, which are crucial for activating CD4+ T cells. This peptide length is well-documented for its effectiveness in MHC Class II binding and presentation, making it a preferred choice for identifying potential epitopes (36–38). The 15-mer peptides are long enough to be processed and presented on the surface of antigen-presenting cells, enhancing their recognition by CD4+ T cells. The predicted HTL epitopes are listed in **Table 2**. These HTL epitopes were subjected to finding the IFN-γ positive HTL epitopes. A total of eight HTL IFN-γ positive epitopes were proposed for the final vaccine, as shown in **Table 2**. The immunogenicity rating of each antigen was then used to choose the top CTL epitopes (refer to **Table 1**). The ABCpred web server was used to forecast the truncated binding score of the epitopes and identify linear B-cell epitopes (refer to **Table 3**). The linear B-cell epitopes with scores >0.9 were chosen as a vaccine candidate. A total of eight HTL gamma positive, 10 CTL, and nine linear B-cell epitopes that passed the allergenicity, antigenicity, and toxicity tests were chosen for final vaccine construction called “S10D16.” HTLs (refer to **Table 2**), cytotoxic T-cell lymphocytes (refer to **Table 1**), and B-cell epitopes (refer to **Table 3**) were linked using the GGPPG, AAY, and KK linkers, respectively. The helper peptide PADRE (AGLFQRHGEGTKATVGEPV) and TLR4 agonist RS-09 (APPHALS) were joined by the EAAAK linker.

### 3.2 Prediction of antigenicity, allergenicity, and toxicity

The VaxiJen v2.0 web tool evaluated the antigenicity of the vaccine construct considered for the vaccine development, and it was predicted to be 0.7086. The VaxiJen v2.0 tool’s antigenicity criterion was set to the default value of 0.4. Both AllergenFP and AllerTOP estimated the vaccine construct *S10D16* to be non-allergen. In addition, ToxinPred predicted the vaccine construct to be non-toxin. It is therefore clear that the developed vaccine is a promising vaccine candidate for the co-infection of dengue and SARS-CoV-2.

### 3.3 Solubility prediction and physicochemical properties

Proteins physicochemical properties play a crucial role in determining their functionality and immunogenicity, particularly in vaccine design. The S10D16 vaccine construct, consisting of 460 amino acids, was analyzed using the Expasy ProtParam service to evaluate these properties. The molecular weight (49,391.51 Da) indicates a size suitable for efficient uptake and processing by APCs, facilitating robust antigen presentation via MHC molecules. The theoretical isoelectric point (pI) of 9.86 suggests that the vaccine construct is positively charged at physiological pH, which enhances its interaction with negatively charged immune cell membranes, such as those of dendritic cells. The elemental formula (C_2203_H_3433_N_643_O_618_S_18_) highlights the presence of sulphur atoms, likely contributing to disulfide bonds that are critical for maintaining the protein’s tertiary structure and stability. The instability index of 39.84 classifies the vaccine as stable, which is essential for preserving structural integrity during storage and administration. Furthermore, the aliphatic index of 63.80 reflects good thermostability, making the vaccine resilient under varying temperature conditions. The GRAVY score of −0.473 indicates a hydrophilic nature, enhancing solubility and bioavailability, which are critical for effective antigen delivery. Additionally, the solubility prediction score of 0.522 confirms that the vaccine has good solubility, a key parameter for ensuring proper formulation and distribution in biological systems. Collectively, these physicochemical properties demonstrate that the *S10D16* vaccine is well suited for stable storage, efficient delivery, and robust immunogenicity, supporting its potential as a reliable multi-epitope vaccine candidate.

### 3.4 Analysis of secondary structure and tertiary structure

**Figure 2:**
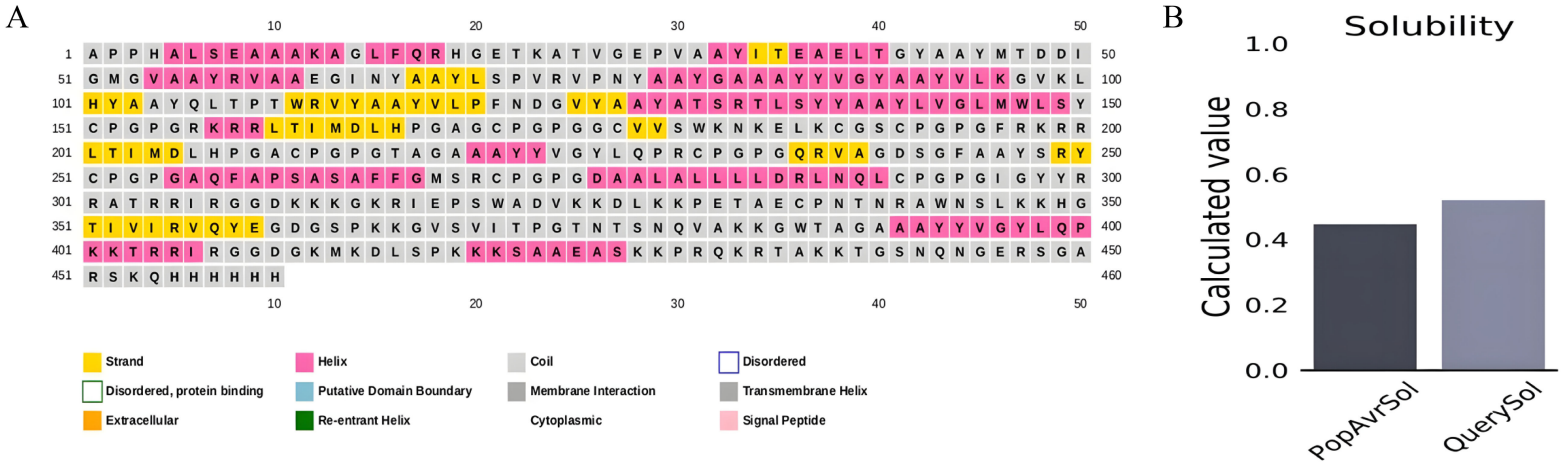
**(A)** Secondary structure prediction using PSIPRED exhibiting 28.48% Alpha helix, 20.65% strand and 50.87% coil. **(B)** ProtSol server solubility analysis showing score of 0.522.

**Figure 3:**
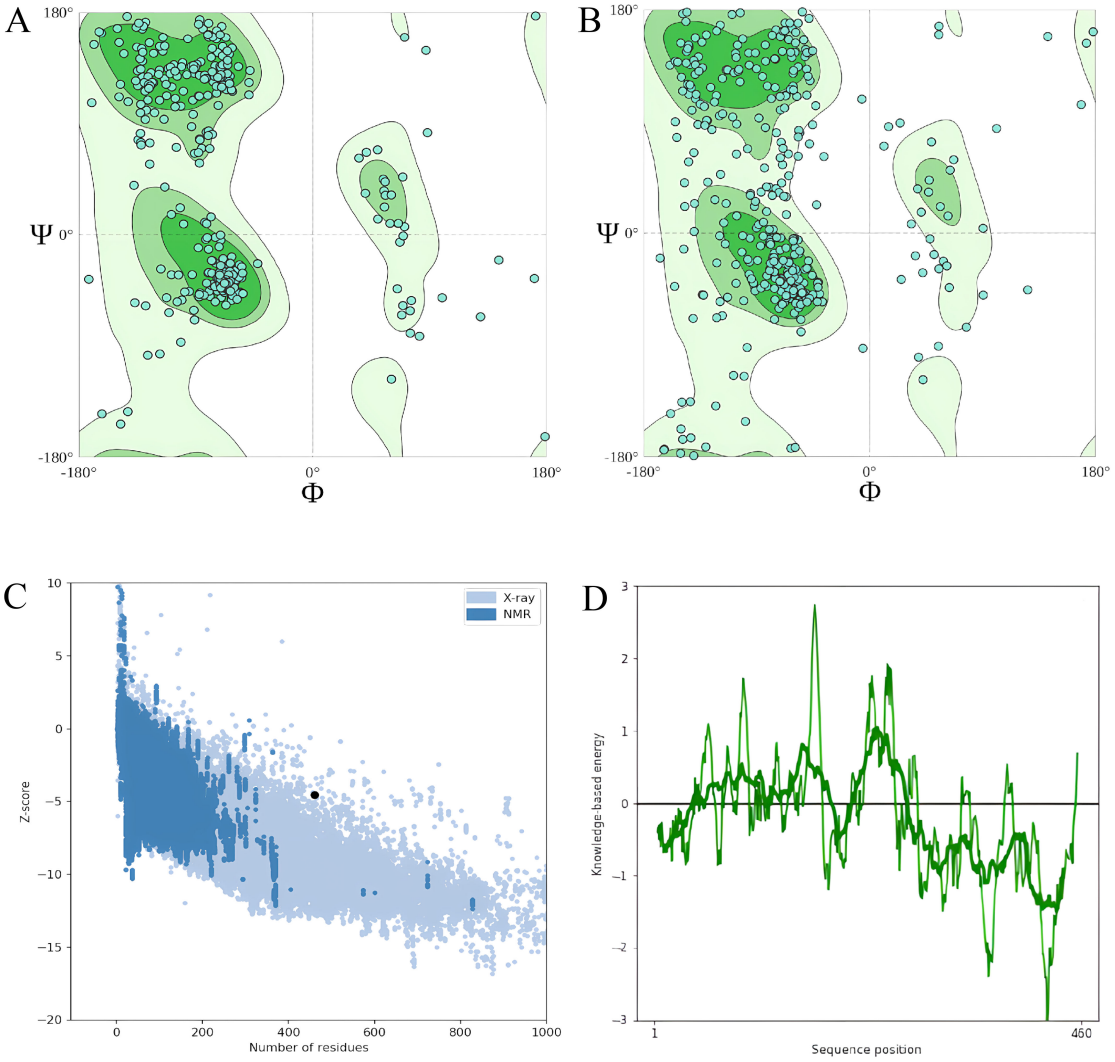
**(A)** The model comprised 65.37% percent preferred regions, 14.40% outlier regions, and 15.00% rotamer regions, according to the Ramachandran plot before optimization. **(B)** The optimized model displayed 85.59%, 3.49%, and 0.88%, in the preferred, outlier, and rotamer sections respectively in the Ramachandran plot. **(C)** *Z*-value score of the optimized vaccine model was −4.56. **(D)** Energy map of the optimized model.

According to estimates, the vaccine sequence as a whole contains 50.87% (234 of 460) coil, 20.65% (95 of 460) strand, and 28.48% (131 of 460) Alpha helix (**Figure 2A**). The ProtSol server solubility analysis obtained a score of 0.522 shown in **Figure 2B**. The I-TASSER web server calculated five tertiary 3D structures with *Z* scores ranging from 0.99 to 2.23 and confidence scores (*C* scores) ranging from −2.03 to −3.07. The modeling’s top structure with a *C*-value of −2.03 was considered for further examination. This structure had a predicted root-mean-square deviation (RMSD) score of 12.3 + −4.3 Å and a likely TM score of 0.46 + −0.15. As a scale for determining how similar the structures are structurally, the TM-value has been suggested. The vaccine’s 3D models were optimized using the GalaxyRefine web server, resulting in significant improvements in the consistency and quality of the candidate proteins. This refinement process enhanced the structural accuracy and reliability of the vaccine design. Five Galaxy server-optimized 3D models were consequently generated. Model 4 was employed for the following studies with a number of benefits, including a GDTHA value of 0.9098, an RMSD value of 0.538 Å, a MolProbity value of 2.454, a Clash score of 17.6, and a poor rotamer of 1.2. Using the ProSA-web and ERRAT-web servers, the quality and significant flaws of the 3D model of the multi-epitope vaccine were examined and validated. The energy map is presented in **Figure 3D**, and the *Z*-value of the optimized vaccine model was −4.56 shown in **Figure 3C**. After optimization with the ERRAT web server, the multi-epitope vaccine’s overall quality factor was 76.334. The model comprised 65.37% preferred regions, 14.40% outlier regions, and 15.00% rotamer regions, according to the Ramachandran plot (**Figure 3A**). Surprisingly, following optimization, the optimized model performed at 85.59%, 3.49%, and 0.88% in the preferred, outlier, and rotamer sections, respectively (**Figure 3B**) (1).

In addition to I-TASSER, we also predicted the structure of the vaccine candidate using Alphafold2 (61) and ESMfold (62). The I-TASSER model was selected over AlphaFold2 and ESMFold due to its superior performance in predicting protein structures with higher reliability and confidence. AlphaFold2 produced low pLDDT values (23.8 to 34.5) and pTM scores (0.136–0.214), indicating poor confidence in the predicted models, while ESMFold also showed weak performance with a mean pLDDT of 29.4 and a pTM score of 0.147. In contrast, I-TASSER generated five tertiary structures with *Z* scores ranging from 0.99 to 2.23 and *C* scores from −2.03 to −3.07, metrics that provide more robust indications of model quality. Among these, the top structure with the highest *C* score (−2.03) was selected for further analysis. This structure had a predicted RMSD of 12.3 ± 4.3 and a TM score of 0.46 ± 0.15, reflecting moderate structural similarity to native proteins. The combination of higher *Z* scores, *C* scores, and TM scores makes I-TASSER the most reliable algorithm in this context, and its top model was deemed the best choice for subsequent applications such as vaccine structure modeling.

### 3.5 Conformational B-cell epitopes

**Figure 4:**
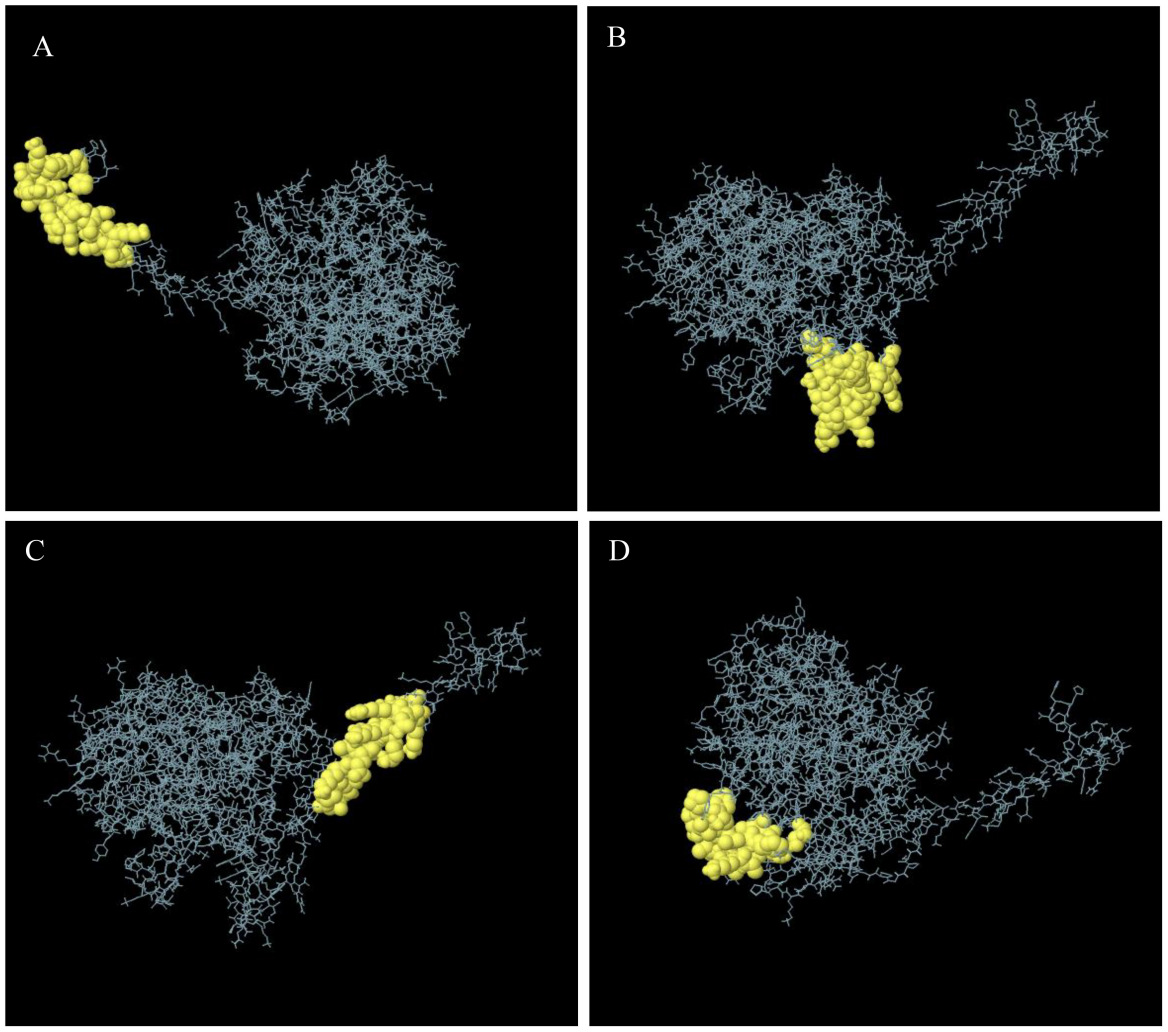
Discontinuous B-cell epitopes 3D representation of the multi-epitope vaccine. **(A–D)** The discontinuous B-cell epitopes are shown by a yellow, and the rest of the polyprotein is shown by gray sticks.

One hundred two-hundred sixty amino acids were predicted to be present in nine discontinuous or conformational B-cell epitopes, ranging from 0.53 to 0.97. The score value of 0.69 or above is typically chosen for discontinuous peptides that Ellipro predicts. Hence, we selected four discontinuous B-cell epitopes with scores greater than 0.69, as shown in **Figure 4**, **Table 4**.

**Table 4:** Conformational or discontinuous B-cell epitope residues and scores.

S.No.	Residues	No. of residues	Score
1	A:T435, A:A436, A:K437, A:K438, A:T439, A:G440, A:S441, A:N442, A:Q443, A:N444, A:G445, A:E446, A:R447, A:S448, A:G449, A:A450, A:R451, A:S452, A:K453, A:Q454, A:H455, A:H456	22	0.97
2	A:P373, A:G374, A:T375, A:N376, A:T377, A:S378, A:N379, A:Q380, A:V381, A:A382, A:K383, A:K384, A:Y394, A:V395, A:G396, A:Y397, A:L398, A:Q399, A:P400, A:K401, A:K402, A:T403, A:R404, A:R405, A:R407	25	0.81
3	A:D415, A:P418, A:K419, A:K421, A:S422, A:A423, A:A424, A:E425, A:A426, A:S427, A:K428, A:K429, A:P430, A:R431, A:Q432, A:K433, A:R434	17	0.76
4	A:A1, A:P2, A:P3, A:H4, A:A5, A:L6, A:S7, A:E8, A:A9, A:A10, A:A11, A:K12, A:A13, A:G14, A:L15, A:F16, A:Q17, A:R18, A:S369	19	0.7

### 3.6 Molecular docking of the S10D16 vaccine constructed with human TLRs

**Figure 5:**
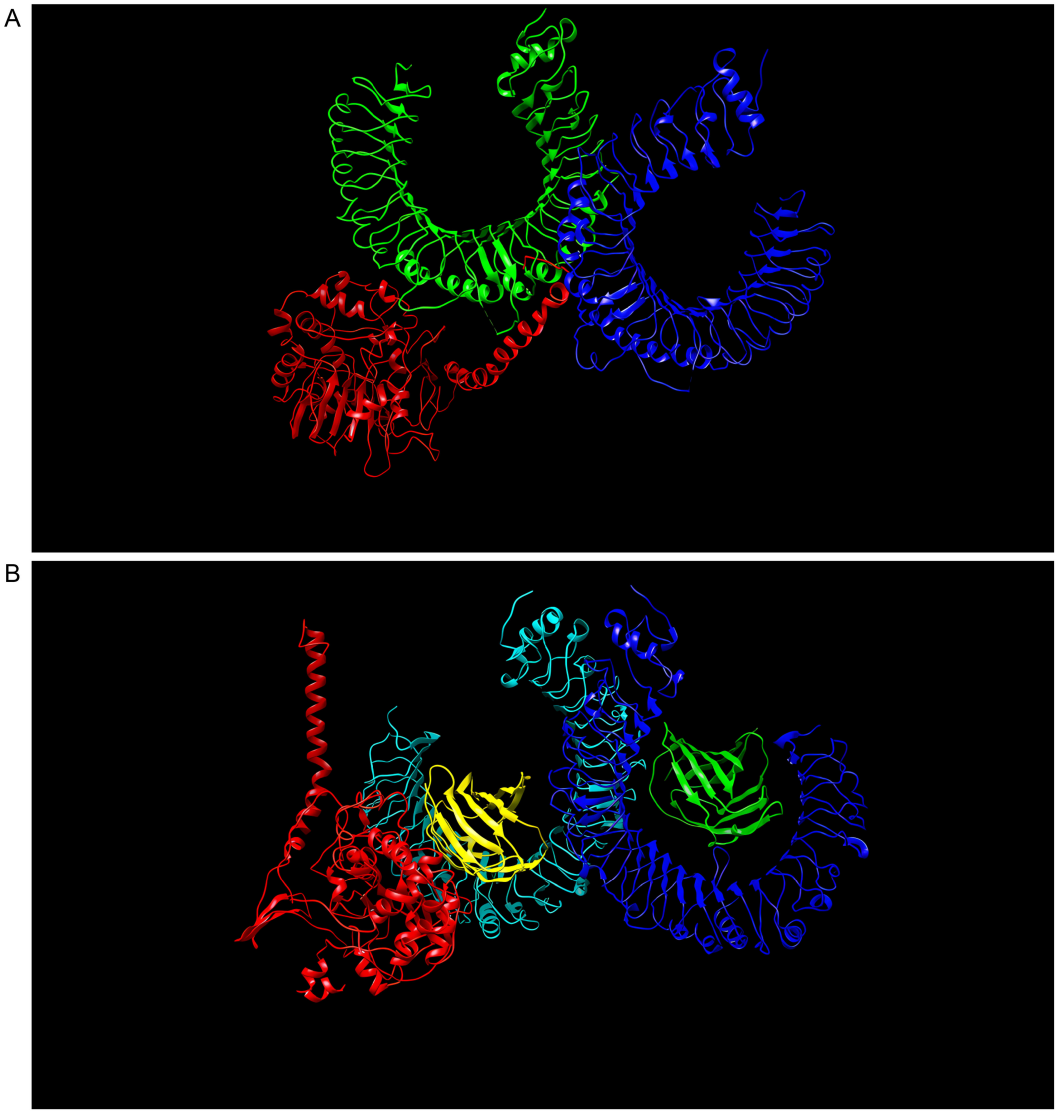
**(A)** Model with lowest binding energy of the “S10D16” vaccine docked with TLR2 performed using ClusPro 2.0. **(B)** Model with lowest binding energy of the “S10D16” vaccine docked with TLR4 performed using ClusPro 2.0.

As a result of docking with either TLR2 or TLR4, the multi-epitope vaccine construct produced 30 models. The TLR2-vaccine docking had a central weighted score of −1041.7 and the lowest energy-weighted score of −1046.8, and the TLR4-vaccine docking had a central weighted score of −974.1 and the lowest energy-weighted score of −1107.9, were chosen for further analysis because of their lower complex binding energies. **Figures 5A, B**, respectively, shows the docking effects of the two models. These findings demonstrated the effectiveness of the multi-epitope vaccine in tightly binding to TLR2 and TLR4 to elicit a potent immunological response.

### 3.7 Normal Mode Analysis dynamics with the vaccine and TLRs

**Figure 6:**
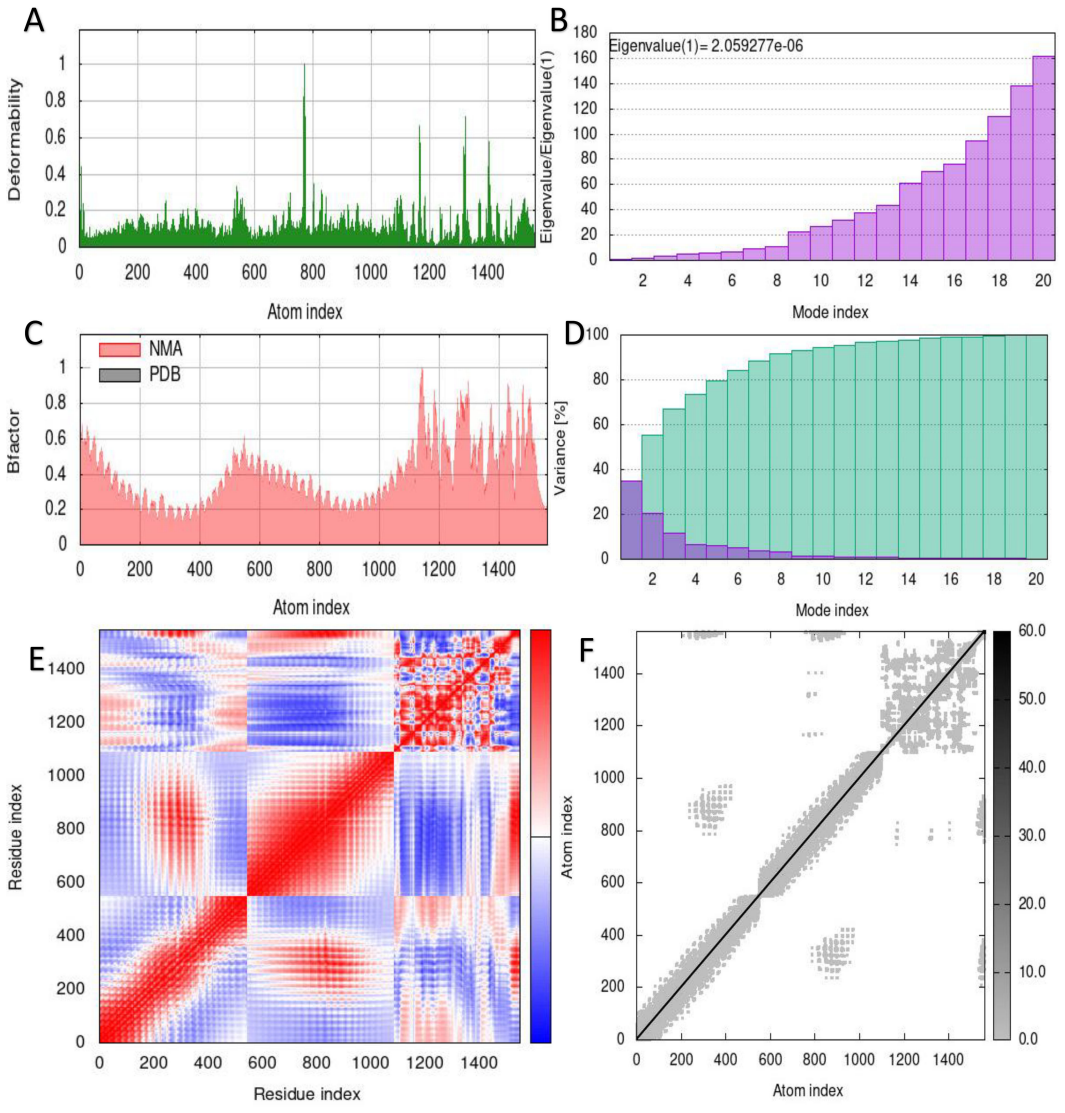
Simulation of the S10D16 vaccine complex using TLR2 and Normal Mode Analysis (NMA) variability **(A)** Deformability plot **(B)** Eigenvalues **(C)** B-factor plot **(D)** A variance plot (Green for cumulative variance, Purple for individual variance **(E)** Covariance plot, correlated motion shown in red and anticorrelated motion shown in blue, respectively **(F)** Network elasticity.

**Figure 7:**
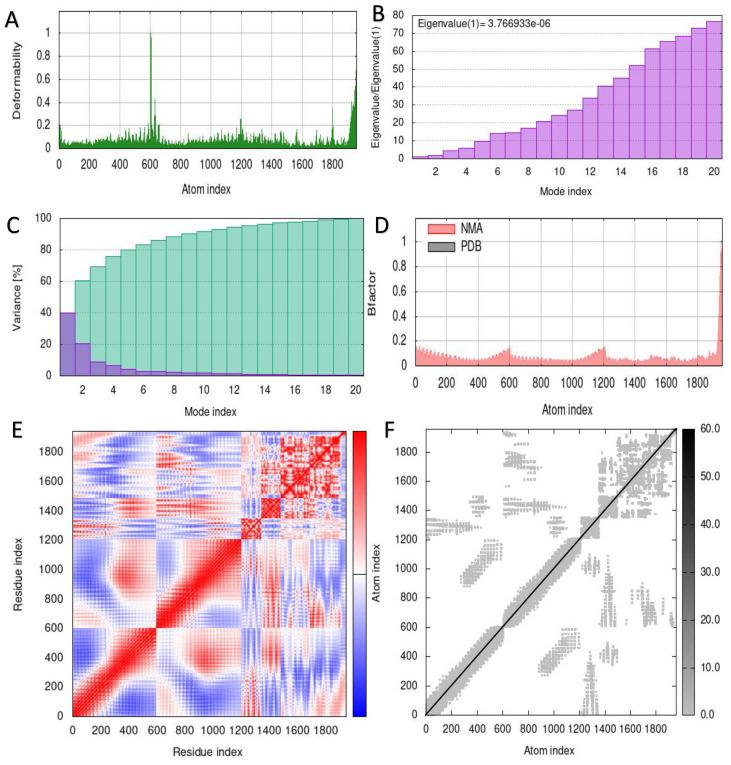
Simulation of the S10D16 vaccine complex using TLR4 and Normal Mode Analysis (NMA) variability **(A)** Deformability plot **(B)** Eigenvalues **(C)** B-factor plot **(D)** A variance plot (Green for cumulative variance, Purple for individual variance **(E)** Covariance plot, correlated motion shown in red and anticorrelated motion shown in blue, respectively **(F)** Network elasticity.

The capacity to predict the motion of atoms and molecules in the antigen structure was predicted through simulations. A distinct peak in the deformable area of the vaccine was visible in the deformability of the TLR2-vaccine and TLR4-vaccine complexes (**Figures 6A**, **7A**). According to **Figures 6B**, **7B**, respectively, TLR2-vaccine and TLR4-vaccine have eigenvalues of 2.059277e-06 and 3.766933e-06. Green and purple covariograms (**Figures 6D**, **7D**) demonstrated cumulative or individual variance, respectively. Docked complexes in the NMA and PDB sectors were related to one another as shown by B-factor graphs (**Figures 6C**, **7C**). The complex’s covariance map describes the relationship among the atoms, with red indicating correlated motion between a pair of residues, white representing uncorrelated motion, and blue representing anti-correlated motion (**Figures 6E**, **7E**); the complex’s elastic map is shown (**Figures 6F**, **7F**).

### 3.8 Immune simulation of the vaccine

**Figure 8:**
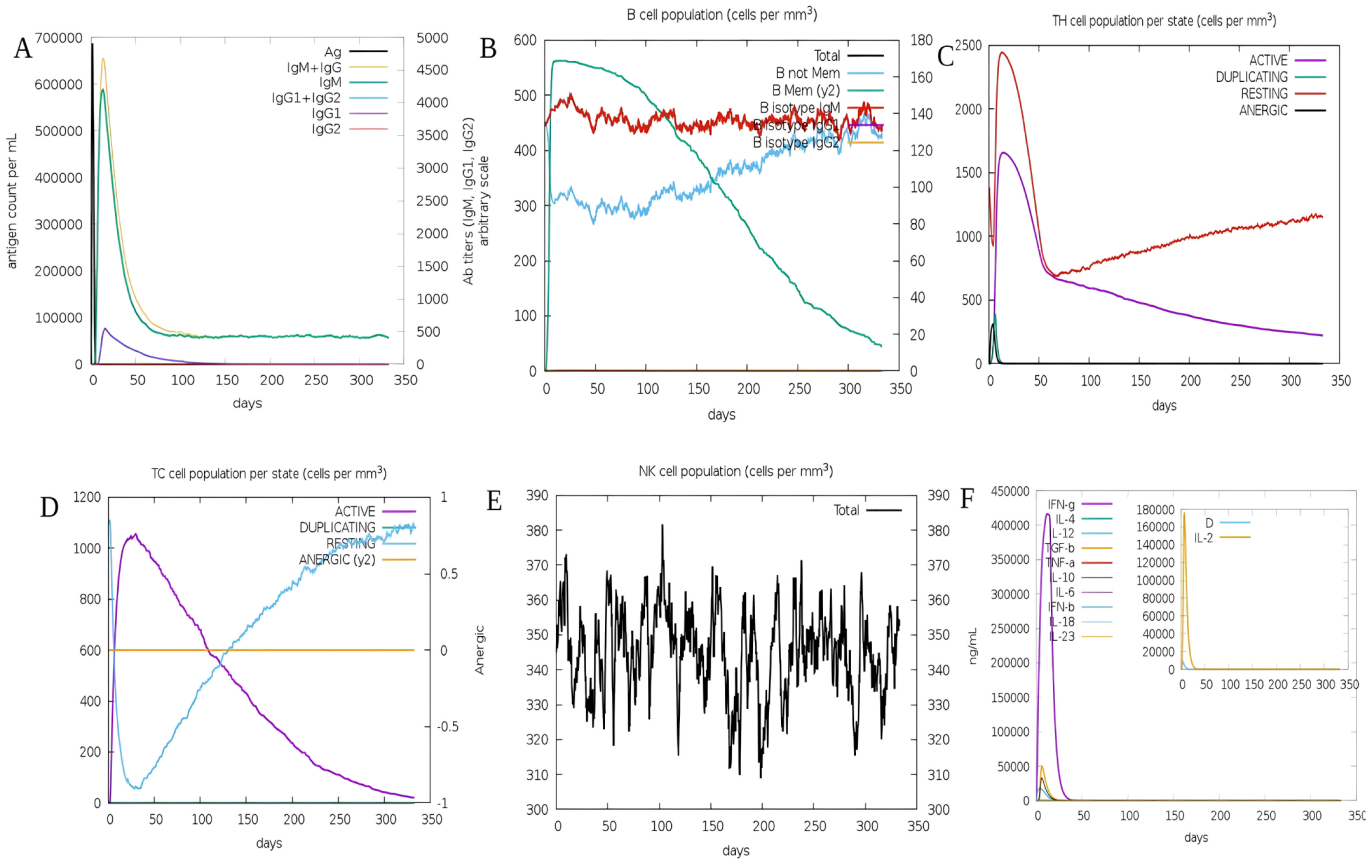
**(A)** B-cell antibodies that are produced after simulation using C-ImmSim **(B)** Changes in B-cell populations **(C)** Changes in HTLs cell populations **(D)** Secretion of CTLs after immune simulation **(E)** NK-cell populations secretions **(F)** Changes in cytokine levels primarily focusing on IFN-γ presented in purple and IL-2 presented in yellow.

Immune responses were simulated using the C-ImmSim server. In the simulation, the vaccine successfully triggered immunological responses and stimulated the innate immune system. According to the findings, the vaccination was able to stimulate B cells to create significant amounts of IgM and IgG antibodies (**Figure 8A**), indicating the development of immunological memory (Lopéz-Blanco, Garzón, and Chacón 2011). T-helper and cytotoxic T-cell populations showed substantial responses and related memory acquisition (**Figures 8C, D**). We discovered that the quantity of active cytotoxic T cells rose progressively and peaked on day 60 following stimulation before starting to drop. The reverse pattern, however, was evident in resting cytotoxic T cells (**Figure 8C**). We also noticed that the *S10D16* vaccination significantly increased the number of B cells that were activated (**Figure 8B**). Additionally, repeated exposure injections at intervals of four weeks following the *S10D16* vaccination caused elevated levels of IFN-γ and IL-2 (**Figure 8F**).

## 4 Discussion

SARS-CoV-2 poses a significant threat to humanity, primarily transmitted through respiratory droplets (1). The methodologies governing the selection and synthesis of biomarkers for vaccine development have diversified and become increasingly data-driven, owing to the rapid advancements in computational biology, bioinformatics, structural biology, and computational software tools (71–73). These innovations have enabled researchers to identify potential targets more efficiently, facilitating the design of vaccines that elicit robust immune responses while minimizing adverse effects.

Various *in-silico* approaches have been attempted to make the multi-epitope vaccine for SARS-CoV-2. For example, Kar et al. proposed a candidate multi-epitope vaccine against SARS-CoV-2 (74). Peele et al. proposed a multi-epitope vaccine is designed using *in silico* tools that potentially trigger both CD4 and CD8 T-cell immune responses against the novel coronavirus (75). Singh et al. designed a multi-epitope peptide-based vaccine against SARS-CoV-2 and also performed immune simulations (76). Ali et al. explored the dengue genome to construct a multi-epitope–based subunit vaccine by utilizing an immunoinformatics approach to battle against dengue infection and evaluated its potential effectiveness through various bioinformatics tools (77). Lim et al. investigate the identification and selection of immunodominant B- and T-cell epitopes for the development of a dengue multi-epitope vaccine (78). These studies highlight the growing interest in leveraging computational methods to design vaccines that can elicit robust immune responses against pathogens.

In this investigation, our objective is to devise a multi-epitope preventive vaccine targeting SARS-CoV-2 and dengue co-infection, constructed utilizing epitopes derived from three distinct SARS-CoV-2 antigens: spike, nucleocapsid, and membrane proteins, alongside one dengue genome polyprotein antigen. This innovative approach aims to enhance the immune response by incorporating conserved epitopes that are critical for both viral infections, thereby increasing the likelihood of cross-protection and reducing the severity of co-infections.

The vaccine design incorporates epitopes from both SARS-CoV-2 and dengue virus to ensure broad representativity and coverage against both pathogens. Specifically, for SARS-CoV-2, the surface spike protein (QHR63290.1) and membrane glycoprotein (QHR63293.1) were chosen due to their crucial roles in virus entry and immune recognition. For the dengue virus, the envelope protein (ID: P29991.1) was selected because it is the primary target for neutralizing antibodies and plays a significant role in viral attachment and fusion (79). The strategy for protection involves the inclusion of multiple T- and B-cell epitopes to elicit a robust and comprehensive immune response. HTL epitopes, cytotoxic T-cell lymphocyte (CTL) epitopes, and B-cell epitopes were predicted and included in the vaccine construct. These epitopes are designed to stimulate both cellular and humoral immunity. HTL epitopes activate CD4+ T cells, which are essential for orchestrating the immune response and providing help to B cells for antibody production. CTL epitopes activate CD8+ T cells, which are crucial for killing infected cells. B-cell epitopes are included to directly stimulate the production of neutralizing antibodies. Neutralizing epitopes are critical for preventing virus entry and infection. For SARS-CoV-2, the spike protein contains known neutralizing epitopes that are targets of neutralizing antibodies produced in response to infection or vaccination (42). For the dengue virus, the envelope protein includes neutralizing epitopes that are the primary targets of the immune response and are crucial for preventing viral entry into host cells (80). By incorporating these epitopes into the vaccine, we aim to induce the production of neutralizing antibodies that can block viral infection.

The final vaccine formulation incorporates the PADRE helper peptide and the TLR4 agonist RS-09, aimed at substantially enhancing its immunogenicity and antigenicity. PADRE serves as a prototypical helper peptide that initiates Th1 cell polarization (81). To augment the immune response elicited by vaccines, the PADRE peptide has the capacity to bind to multiple MHC-II allele types (82). Our results illustrate the vaccine’s consistent affinity for TLR2 and TLR4, laying the essential foundation for the immunization process to recognize and activate TLR signaling pathways. A vaccine must possess robust immunogenicity and antigenicity, exhibit non-toxic properties, and avoid inducing allergic reactions. Ideally, it should be developed employing bioinformatics and immunoinformatics methodologies. Furthermore, it should elicit a potent immune response while mitigating adverse side effects. Our results indicate that the constructed vaccine displays strong antigenic properties, is non-toxic, and is devoid of allergenic characteristics, thereby positioning it as a promising candidate for addressing SARS-CoV-2 and dengue co-infection. We successfully linked epitopes utilizing EAAAK, GPGPG, AAY, and KK linkers, which are integral components in vaccine development and facilitate expression, proper folding, and stability (83). For instance, the AAY linker enhances the stability of the associated structures by providing proteasome cleavage sites (84). Additionally, KK linkers can aid in maintaining the independent immunogenic activity of epitopes (85). Ultimately, we were able to delineate the complete sequencing and structural composition of the vaccine. The vaccine design comprises 460 residues, exhibiting a molecular weight of 49391.51 Da, a theoretical isoelectric point of 9.86, a lipid index of 39.84, a GRAVY score of −0.473, an aliphatic index of 63.80, and an instability index of 39.84. Based on these parameters, the vaccine is classified as stable. Collectively, the vaccine possesses attributes that render it a compelling candidate for subsequent *in-vivo* evaluations. Multi-epitope vaccines are adept at eliciting immune responses to specific epitopes while avoiding the induction of systemic autoimmunity (53). In this study, we included both T- and B-cell epitopes, thereby facilitating both cellular and humoral immune responses. We significantly mitigated the potential adverse effects associated with non-essential epitopes arising from complete protein antigens by opting not to include the entire protein. This strategy is anticipated to optimize the establishment of protective and targeted immune responses (86). Our immune stimulation assays corroborated this assertion, as the vaccine successfully elicited robust cellular and humoral immunity. In this research endeavor, we also undertook predictions concerning the IFN-γ release score of HTL epitopes to identify those that positively correlate with IFN-γ (87). A notable limitation of numerous existing neutralizing antibodies is their incapacity to confer enduring immunity and protection to individuals lacking T-cell immunity. Thus, the critical importance of T-cell immunity is unequivocal (88). In conclusion, the *S10D16* vaccine demonstrates promising biological characteristics and structural properties, meriting further exploration through both *in-vitro* and *in-vivo* validation. Continuous optimization efforts are essential to ensure that this vaccine becomes a dependable intervention for preventing co-infections involving both Dengue and SARS-CoV-2. However, it is crucial to recognize that this study has several limitations. First, the evaluation of the vaccine’s physicochemical and immunological properties was exclusively conducted through *in-silico* methods, lacking corroboration via *in-vitro* and *in-vivo* studies. Second, only four antigens were selected for the purpose of predicting and screening immunodominant epitopes, which may have constrained the vaccine’s efficacy in preventing the co-infection of dengue and SARS-CoV-2.

In this study, we demonstrated the potential of combining epitopes from both dengue and SARS-CoV-2 to enhance cross-protection, suggesting a novel avenue for vaccine development that warrants further exploration. It can be provided to people who are at high risk of severe outcomes from these infections, particularly in regions where both viruses are endemic. Additionally, the integration of adjuvants may further amplify the immune response, making it crucial to evaluate their effects in clinical trials. However, it is important to acknowledge that this study does have several limitations. Firstly, the assessment of the vaccine’s physicochemical and immunological characteristics was solely conducted *in silico*, lacking validation through *in-vitro* and *in-vivo* experiments. Second, only four antigens were chosen for the purpose of predicting and screening immunodominant epitopes, which may have reduced the vaccine’s ability to prevent the co-infection of dengue and SARS-CoV-2.

## 5 Conclusions

In the present investigation, a novel multi-epitope vaccine called “*S10D16*” was developed, a novel and innovative multi-epitope vaccine tailored for targeting both dengue and SARS-CoV-2, demonstrating potential as a dual-action immunization strategy. It has been designated with the name “*S10D16*” by integrating a total of eight distinct HTL epitopes, ten specific CTL epitopes, and nine unique B-cell epitopes, in addition to the PADRE sequence and the component known as RS-09. Our comprehensive research elucidates and clarifies that this vaccine exhibits a number of advantageous attributes concerning its antigenicity, along with its non-toxicity and non-allergenicity, making it a suitable candidate for further exploration. It has convincingly demonstrated the remarkable ability to effectively elicit vigorous and robust immune responses while simultaneously circumventing any potential deleterious effects that could arise from its administration. This thorough investigation presents what appears to be a highly promising vaccine candidate that is specifically aimed at mitigating the challenges posed by co-infection associated with the viruses SARS-CoV-2 and dengue, thereby offering innovative and strategic approaches for significantly reducing the global transmission rates of these infectious viruses. The study’s limitations include solely *in-silico* assessment of vaccine characteristics without *in-vitro* or *in-vivo* validation.

## Data Availability

The original contributions presented in the study are included in the article/supplementary material. Further inquiries can be directed to the corresponding authors.
